# Both sides of the bell curve: Base rates of high and low scores in cognitively unimpaired and impaired older adults and their relationship to biomarkers of Alzheimer’s disease

**DOI:** 10.1017/S1355617725101227

**Published:** 2025-08-22

**Authors:** Kevin Duff, Chase Presley, Jace B. King, John M. Hoffman, Rune Raudeberg

**Affiliations:** 1 Layton Aging and Alzheimer’s Disease Center, Department of Neurology, Oregon Health & Science University, Portland, OR, USA; 2 Center for Alzheimer’s Care, Imaging and Research, Department of Neurology, University of Utah, Salt Lake City, UT, USA; 3 Department of Neurology, Vanderbilt University Medical Center, Nashville, TN, USA; 4 Department of Radiology and Imaging Sciences, University of Utah, Salt Lake City, UT, USA; 5 University of Utah Center for Quantitative Cancer Imaging, Huntsman Cancer Institute, Salt Lake City, UT, USA; 6 Department of Biological and Medical Psychology, University of Bergen, Bergen, Norway

**Keywords:** Assessment, base rates, neuropsychological tests, cognitive aging, psychometrics, biomarkers

## Abstract

**Objective:**

To further investigate the “other side of the bell curve” hypothesis, the current study examined the number of low and high scores on a neuropsychological battery: 1) in cognitively unimpaired or impaired older adults, 2) as they relate to biomarkers of Alzheimer’s disease (AD), and 3) as they relate to traditional scores on this battery.

**Method:**

In 68 cognitively unimpaired and 97 cognitively impaired participant, the number of low (i.e., ≤ 16^th^ percentile) and high (i.e., ≥ 75^th^ percentile) scores on the Repeatable Battery for the Assessment of Neuropsychological Status (RBANS) were calculated, compared between the two groups, and related to biomarkers of AD (i.e., amyloid deposition, hippocampal volumes, ε4 alleles of Apolipoprotein E (APOE)) and RBANS Total score.

**Results:**

In this cognitively diverse sample, low and high scores were common, with approximately 75% having at least one low score and 86% having at least one high score. Unimpaired participants had significantly more high scores and fewer low scores than their impaired counterparts. The number of low scores was significantly related to more amyloid deposition, smaller hippocampal volume, and having one or more copies of the ε4 allele of APOE. The number of high scores was similarly related with these biomarkers. Low/high scores were comparable to traditional scores on the RBANS in identifying cognitively impaired participants.

**Conclusions:**

Support for the “other side of the bell curve” hypothesis was equivocal in these analyses, with both sides of the bell curve appearing to provide relevant information in a cognitively diverse sample.

## Introduction

Whereas the presence of low scores on neuropsychological tests are commonly examined to identify cognitive impairment in older adults, Karr and colleagues (2020) have noted that the absence of high scores may also provide valuable clinical information, especially in identifying subtle cognitive weaknesses in high functioning individuals. They deemed this hypothesis the “other side of the bell curve,” as they initially examined the multivariate base rate of high scores, which could be used to identify individuals who met or failed to meet expectations of high scores. For example, they used standardization data from the Delis–Kaplan Executive Function System to calculate base rates of high scores (e.g., > 74^th^ percentile). They also provided two case examples to suggest that the absence of high scores could indicate a cognitive deficit in high functioning individuals. Using a subset of the normative sample for the National Institutes of Health Toolbox for the Assessment of Neurological and Behavioral Function Cognition Battery, Iverson & Karr (2021) reported base rates of low (e.g., ≤ 25^th^ percentile) and high (e.g., ≥ 75^th^ percentile) scores. They also developed algorithms to identify cognitive impairment using these base rates, which they suggested would be seen by the absence of high scores in high functioning individuals. More recently, Karr et al. (2024) demonstrated that individuals with subjective cognitive concerns presented with a similar number of low scores but fewer high scores than individuals without such concerns. Although their operational definitions varied slightly across studies, these authors have largely described high functioning individuals as those who scored ≥ 75^th^ percentile on a measure of premorbid/crystalized intellect and/or obtained a college degree.

Although intriguing as a method to detect subtler cognitive difficulties in individuals before they cross typical thresholds for impairment, this “other side of the bell curve” hypothesis needs additional validation before it might be ready for clinical practice. For example, in the existing studies on this method, only cognitively unimpaired samples have been examined. However, Karr and colleagues (Iverson & Karr, 2021; Karr et al., 2020; Karr & Iverson, 2020) have recommended that future studies of this hypothesis examine if the absence of high scores could be used to detect cognitive decline in high functioning cognitively impaired individuals. For the clinical utility of this hypothesis to be supported, cognitively high functioning individuals with late life cognitive disorders should present with few/no high scores.

Additionally, there has been relatively little presented on the relationship of the presence/absence of high scores and biomarkers of neurological disease (e.g., amyloid deposition or brain atrophy typically seen in Alzheimer’s disease [AD]). Karr et al. (2024) did examine regional brain volume, cortical thickness, and connectivity of the frontoparietal control network in older adults with and without subjective cognitive complaints and their low and high scores on a battery of memory and executive functioning tests. In that study, none of these neuroimaging variables were significantly related to the number of low or high scores.

Therefore, the current study sought to further investigate the number of low and high scores on a neuropsychological battery in four ways. First, the presence or absence of low and high scores on the Repeatable Battery for the Assessment of Neuropsychological Status (RBANS) were examined in older adults, which have not yet been examined. Second, these low and high scores were examined in individuals who were either cognitively unimpaired or impaired, with the hypothesis that those who were cognitively impaired would show more low scores and fewer high scores than those who were cognitively unimpaired. Third, relationships were evaluated between low and high scores and three common biomarkers of AD (amyloid deposition on a positron emission tomography [PET], hippocampal volumes on magnetic resonance imaging [MRI], ε4 alleles on Apolipoprotein E [APOE testing]). Support for the “other side of the bell curve” hypothesis would be that these biomarkers would be more common in individuals who had few high scores (and less common in those with many high scores). Finally, these low and high scores were compared to more traditional scores on the RBANS (e.g., Total Scale score). Support for this hypothesis would show that low and/or high scores correctly identified more participant as either cognitively intact or impaired than the traditional scores on the RBANS.

## Method

### Participants

One hundred sixty-five older adults were recruited from a cognitive disorders’ clinic (53.6%) or through the community (46.4%) to participate in a larger study of brain imaging and neuropsychological testing across the dementia spectrum. Data was collected between 2018–2022. Their mean age was 74.1 (SD = 6.0) years and their mean education was 16.0 (SD = 2.5) years. Most were Caucasian (98.2%) and 56.6% were female. Mean premorbid intellectual functioning – as measured by the Reading subtest of the Wide Range Achievement Test–4 (WRAT-4) – was in the average to high average range (*M* = 110.2, SD = 8.4), and self-rating of depression symptoms were minimal on the 15-item Geriatric Depression Scale (GDS, *M* = 1.3, SD = 1.3). Consistent with the work of Karr and colleagues in this area, 90% of the sample was classified as “high functioning” (i.e., ≥ 75^th^ percentile on a WRAT-4 Reading and/or completion of a college degree).

Participants from the cognitive disorders’ clinic were recruited with a clinical diagnosis of either amnestic Mild Cognitive Impairment (MCI; single or multi-domain) or AD based on a neurological visit, neuropsychological evaluation, and brain imaging. Participants from the community were largely recruited as cognitively unimpaired controls; however, a minority of amnestic MCI and AD cases (13% and 12%, respectively) were identified in the community. Confirmation of group assignment was made with the Alzheimer’s Disease Neuroimaging Initiative classification battery (Petersen et al., 2010), which included the Mini Mental Status Examination (Folstein et al., 1975), the Clinical Dementia Rating Scale (Morris, 1993), and the Wechsler Memory Scale – Revised (Wechsler, 1987) Logical Memory II Paragraph A. Based on these criteria, 68 participants were classified as cognitively unimpaired, 52 as amnestic MCI (single or multidomain), and 45 as mild AD.

Participants were included if they were 65 years of age or older and had a knowledgeable collateral source available to comment on their cognition and daily functioning. Participants were excluded for medical comorbidities likely to affect cognition (including neurological conditions, substance abuse, and major psychiatric conditions), the inability to complete MRI or positron emission tomography (PET), the inability to complete cognitive assessments due to inadequate vision, hearing, or manual dexterity, and being enrolled in a clinical drug trial related to anti-amyloid agents. Additional exclusion criteria included elevated depression as indicated by a score of greater than 5 on the GDS, and moderate or severe dementia as indicated by a Clinical Dementia Rating score of 2 or greater or a Mini Mental Status Examination score of less than 20. No additional attempts were made to limit the sample or control any potential biases.

### Procedure

Procedures were approved by the Institutional Review Board at the University of Utah and in accordance with the Helsinki Declaration before participants enrolled. Following informed consent/assent, participants underwent testing with the Alzheimer’s Disease Neuroimaging Initiative battery and other neuropsychological testing at a baseline visit. They returned about a week later (*M* = 6.9 days, SD = 0.7) for an MRI of the brain. Within about a month (*M* = 36.4 days, SD = 49.0), they returned to receive an amyloid PET scan of the brain using ^18^F-Flutemetamol and a blood draw to determine APOE ε4 status.

### Neuropsychological measures

The following neuropsychological tests were administered at a baseline visit. These test results were neither part of the clinical evaluation of individuals nor of the research classification that confirmed group assignment (i.e., they were independent of any diagnosis).

- GDS – 15-item version (Yesavage et al., 1982) is a self-report scale of depressive symptoms experienced over the past week. Higher raw scores indicate more depressive symptoms.
- The WRAT-4 Reading subtest (Wilkinson & Robertson, 2006), in which a participant reads irregular words, was administered to assess premorbid intellect. Using normative data, age-corrected standard scores are generated (*M* = 100, SD = 15), with higher scores indicating higher premorbid intellect.
- The RBANS (Randolph, 2012) is a brief neuropsychological testing battery comprised of 12 subtests: List Learning, Story Memory, Figure Copy, Line Orientation, Picture Naming, Semantic Fluency, Digit Span, Coding, List Recall, List Recognition, Story Recall, and Figure Recall. Using regression-based normative data that was corrected for age, education, sex, and race (Duff & Ramezani, 2015), each subtest score was converted to a z-score, with higher scores indicating better cognition. These 12 subtests are combined to form an overall measure of current cognition, the Total Scale score, which are age-corrected standard scores (*M* = 100, SD = 15) based on the test manual.

### Amyloid imaging

Amyloid imaging was performed using ^18^F-Flutemetamol which is a radioactive diagnostic agent indicated for PET imaging of the brain to estimate beta-amyloid neuritic plaque density in adult patients with cognitive impairment. ^18^F-Flutemetamol was produced under PET current Good Manufacturing Practice standards and conducted under an approved Food and Drug Administration Investigational New Drug application. Twenty minutes of emission imaging was performed 90 minutes after the injection of approximately 185 mBq (5 mCi) of ^18^F-Flutemetamol. A GE Discovery PET/CT 710 (GE Healthcare) was used in this study. This PET/CT scanner has full width at half-maximum spatial resolution of 5.0 mm and excellent performance characteristics (Sunderland & Christian, 2015; Yester et al., 2014). Volumes of interest were automatically generated by using the CortexID Suite analysis software (GE Healthcare). ^18^F-Flutemetamol binding was analyzed using a regional semi-quantitative technique described by Vandenberghe et al. (2010) and refined by Thurfjell et al. (2014). The CortexID Suite software generates, semi-quantitative regional (prefrontal, anterior cingulate, precuneus/posterior cingulate, parietal, mesial temporal, lateral temporal, occipital, sensorimotor, cerebellar gray matter, and whole cerebellum) standardized uptake value ratios normalized to the pons. A composite standardized uptake value ratio (SUVR) in the cerebral cortex was generated automatically and normalized to the pons using the CortexID Suite software (Lundqvist et al., 2013).

### MRI

MRI was acquired on a 3.0-T Siemens Prisma scanner with a 64-channel head coil. Structural data were acquired using an MP2RAGE sequence (TR = 5000, TE = 2.93, acquired sagittally, resolution = 1 × 1 × 1 mm) to obtain high quality, whole brain 1 mm isotropic T1 images with improved signal homogeneity in ~ 7 minutes. All MRI scans were examined for the presence of common artifacts, including motion, susceptibility, and distortion, and were determined to be of sufficient quality for quantitative analysis. All data were processed on the same workstation using FreeSurfer image analysis suite v6.0 (http://surfer.nmr.mgh.harvard.edu/) to estimate total estimated intracranial and hippocampal volumes. Technical details are described previously (Fischl & Dale, 2000; Fischl et al., 2002; Fischl et al., 2004). To address head size differences, hippocampal volumes were expressed as a proportion of the estimated total intracranial volume. Left and right hemispheric volumes were summed to create total hippocampal volume adjusted by total intracranial volume. Hippocampal volume data was missing on 4 unimpaired and 9 impaired participants. Although some of this was due to technical problems with data collection (e.g., movement artifact), some was due to characteristics of the participants (e.g., claustrophobia, significant atrophy). As such, there is likely some bias in the results using the hippocampal volumes.

### APOE genotyping

Polymerase Chain Reaction and Fluorescence Monitoring using hybridization probes for APOE genotyping was conducted using whole blood samples. The number of APOE ε4 alleles was determined for each participant as none, one, or two.

### Data analysis

For each participant, each of the 12 RBANS subtest was categorized as a “low” score if it was at or below the 16^th^ percentile (i.e., z-score ≤ −1.00) or as a “high” score if it was at or above the 75^th^ percentile (i.e., z-score ≥ 0.67), which is consistent with prior work in this area (Karr et al., 2024). The number of “low” scores were summed, and the number of “high” scores were summed, with each ranging from 0 (i.e., no low/high scores)–12 (i.e., all low/high scores). For the current analyses, the MCI and AD participants were combined into a cognitively impaired group (*n* = 97), who were compared to those in the cognitively unimpaired group (*n* = 68). Although the summed number of “low” and “high” scores were fairly normally distributed in the entire sample, they were not normally distributed (e.g., skewness > 3) in the two subgroups (cognitively unimpaired and cognitively impaired). As such, nonparametric statistics were used throughout the primary and secondary analyses.

Three primary analyses were examined. First, to examine if low/high scores were more common in cognitively unimpaired or impaired participants, these two groups were compared on the summed low/high scores with Mann–Whitney U tests. Second, to examine if these low/high scores were associated with amyloid deposition and hippocampal volumes in the entire sample, Spearman correlations were calculated between the summed low/high scores and (1) the composite SUVR and (2) the total hippocampal volume. To examine if these low/high scores were associated with APOE ε4 in the entire sample, independent samples Kruskal–Wallis tests compared these three groups (i.e., no, one, or two ε4 alleles) on the summed low/high scores. Third, to examine how the low/high scores compared to traditional scores on the RBANS, receiver operating characteristic curves were generated for low scores, reversed high scores, and reversed RBANS Total Scale scores, for all subjects, where a positive score reflected a cognitively impaired individual. High and RBANS Total scores were reversed so that all three scores went in the same direction for impaired individuals. In secondary analyses, associations between low/high scores and biomarkers were calculated on each of the two cognitive groups (i.e., unimpaired and impaired). The sample size was based on available data, and no a priori power calculations were completed to estimate sample size. As noted above, missing data was only present on hippocampal volumes, but missing data did not restrict analyses. An alpha level of .05 was used throughout, but exact *p*-values are reported for all analyses. Given the exploratory nature of these analyses (i.e., re-analysis of existing data not designed to answer these specific research questions), no correction for multiple comparisons were made.

## Results

### Preliminary analyses

The 68 cognitively unimpaired subjects were significantly younger (t[166] = 2.6, *p* = .01) and better educated (t[166] = 2.1, *p* = .03) than the 97 cognitively impaired individuals. There were no differences between the groups in sex (*p* = .53), race (*p* = .68), or premorbid intellect (*p* = .12). However, since the normative data corrected for age, education, sex, and race, these demographic differences were not considered as covariates in the primary analyses. Ninety percent of the sample was found to be “high functioning,” and a higher percentage of high functioning individuals were cognitively unimpaired (98%) rather than cognitively impaired (85%, c^2^[1] = 8.9, *p* = .003).

### Frequency of low/high scores

In the entire sample, low scores (i.e., ≤ 16^th^ percentile) were quite common (Table 1). In fact, approximately 75% of the sample had at least one low score, approximately 50% had three or more low scores, and approximately 25% had six or more low scores. The mean number of low scores for the cohort was 3.73 (SD = 3.30, range = 0–12) out of 12 possible scores on the RBANS. Similarly, high scores (i.e., ≥ 75^th^ percentile) were prevalent in the entire sample (Table 1), with 86% having at least one high score. The mean number of high scores for this group was 3.01 (SD = 2.63, range = 0–10) out of 12 scores. In these 165 subjects, the number of low scores was significantly and negatively related to the number of high scores (ρ=−.80, *p* < .001).

When comparing the cognitively unimpaired participants to the cognitively impaired participants, there were differences on both sets of scores (see Table 1). Not surprisingly, the unimpaired participants had significantly more high scores than their impaired counterparts (U[N = 165] = 666.5, *p* < .001, *r* = .69). Also not surprisingly, the impaired participants had significantly more low scores than their unimpaired peers (U[N = 165] = 6316.5, *p* < .001, *r* = .79). As in the entire sample, there were significant and negative relationships between the number of low scores and high scores in each subgroup, although they tended to be smaller than in the entire sample (unimpaired: ρ=−.38, *p* = .001; impaired: ρ=−.60, *p* < .001).

### Association with biomarkers

**Figure 1.**
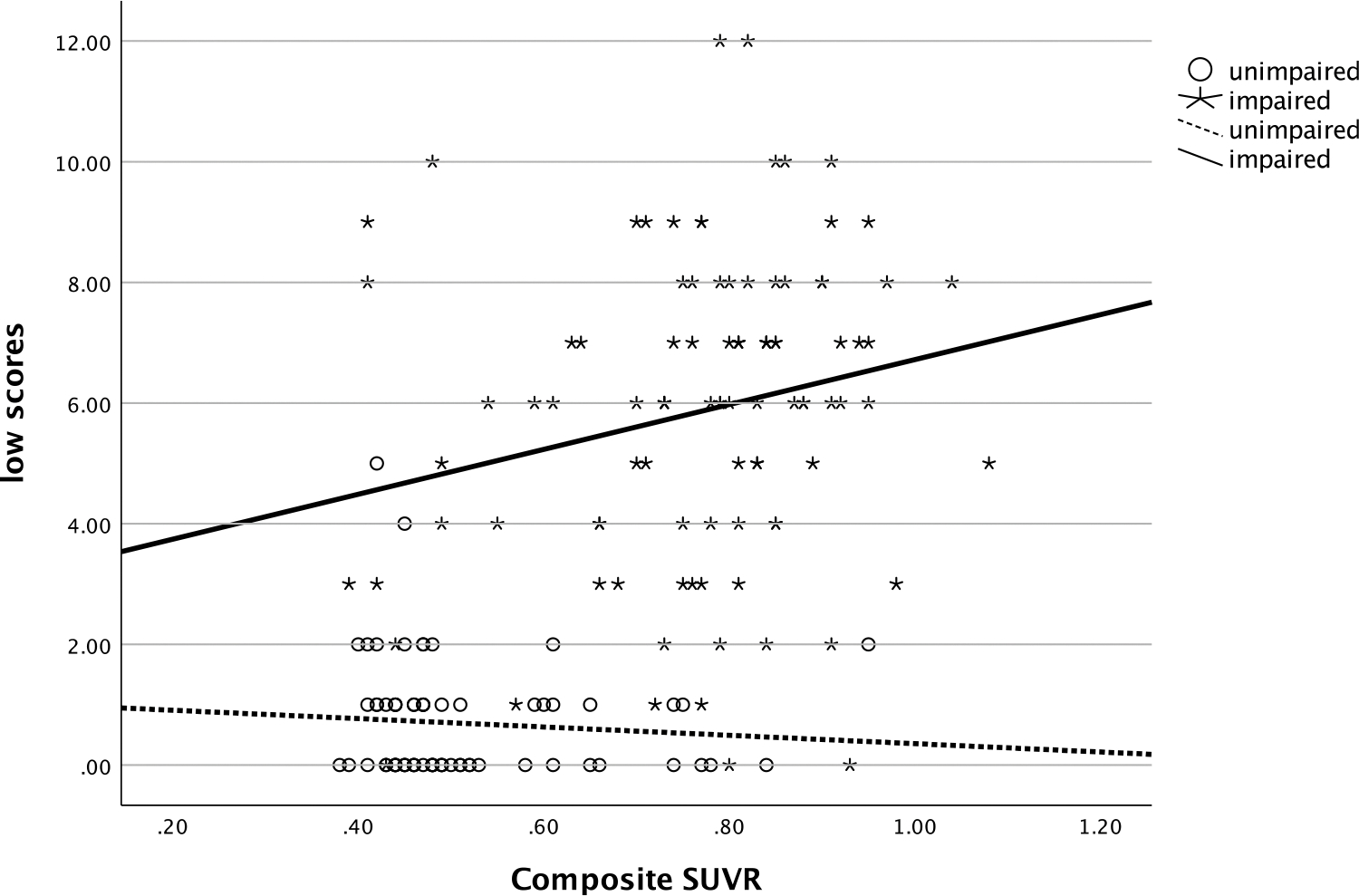
Relationship of number of low scores and amyloid deposition. *Note*: low scores = number of scores ≤ 16^th^ percentile; SUVR = standardized uptake value ratio.

**Figure 2.**
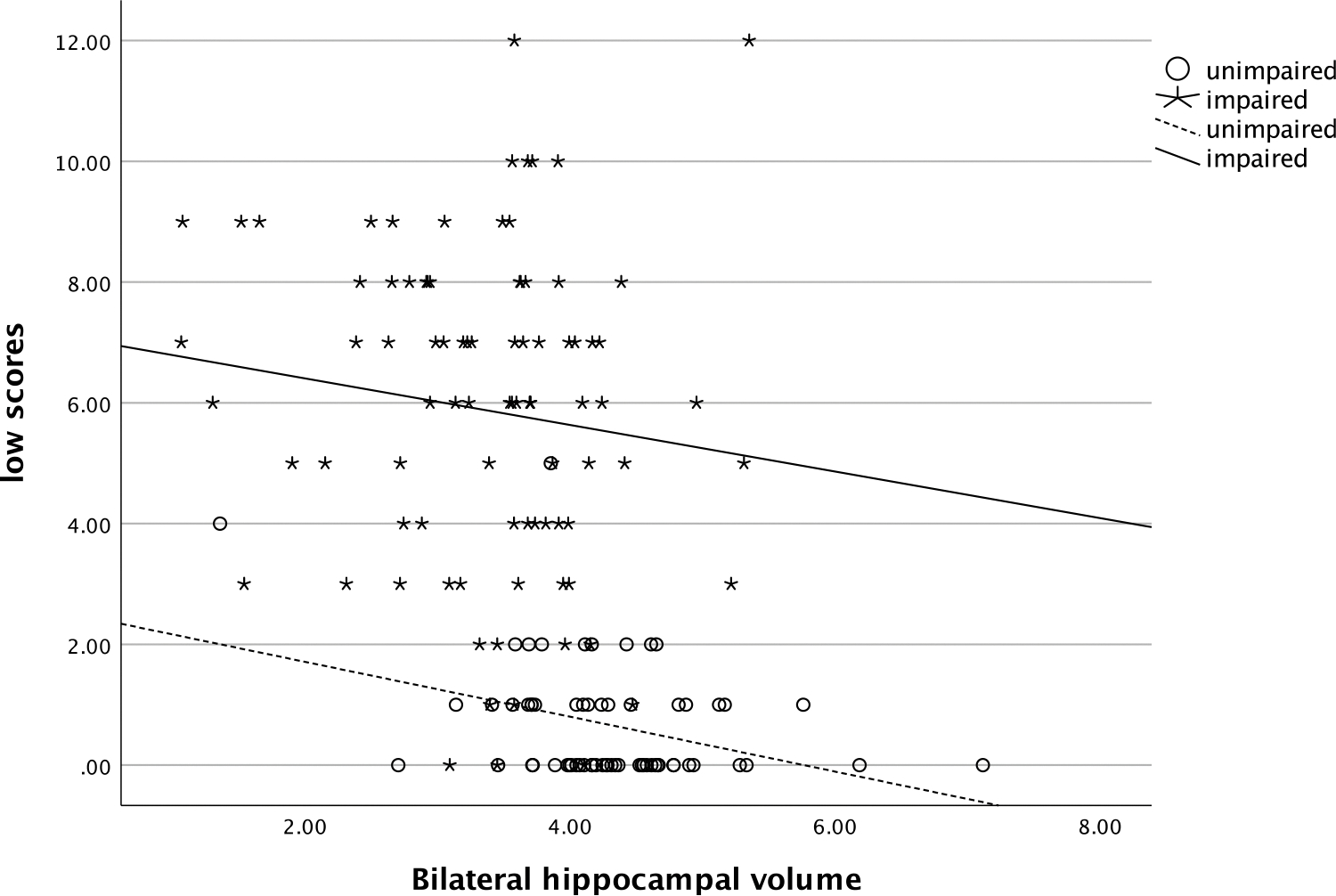
Relationship of number of low scores and bilateral hippocampal volume. *Note*: low scores = number of scores ≤ 16^th^ percentile.

**Figure 3.**
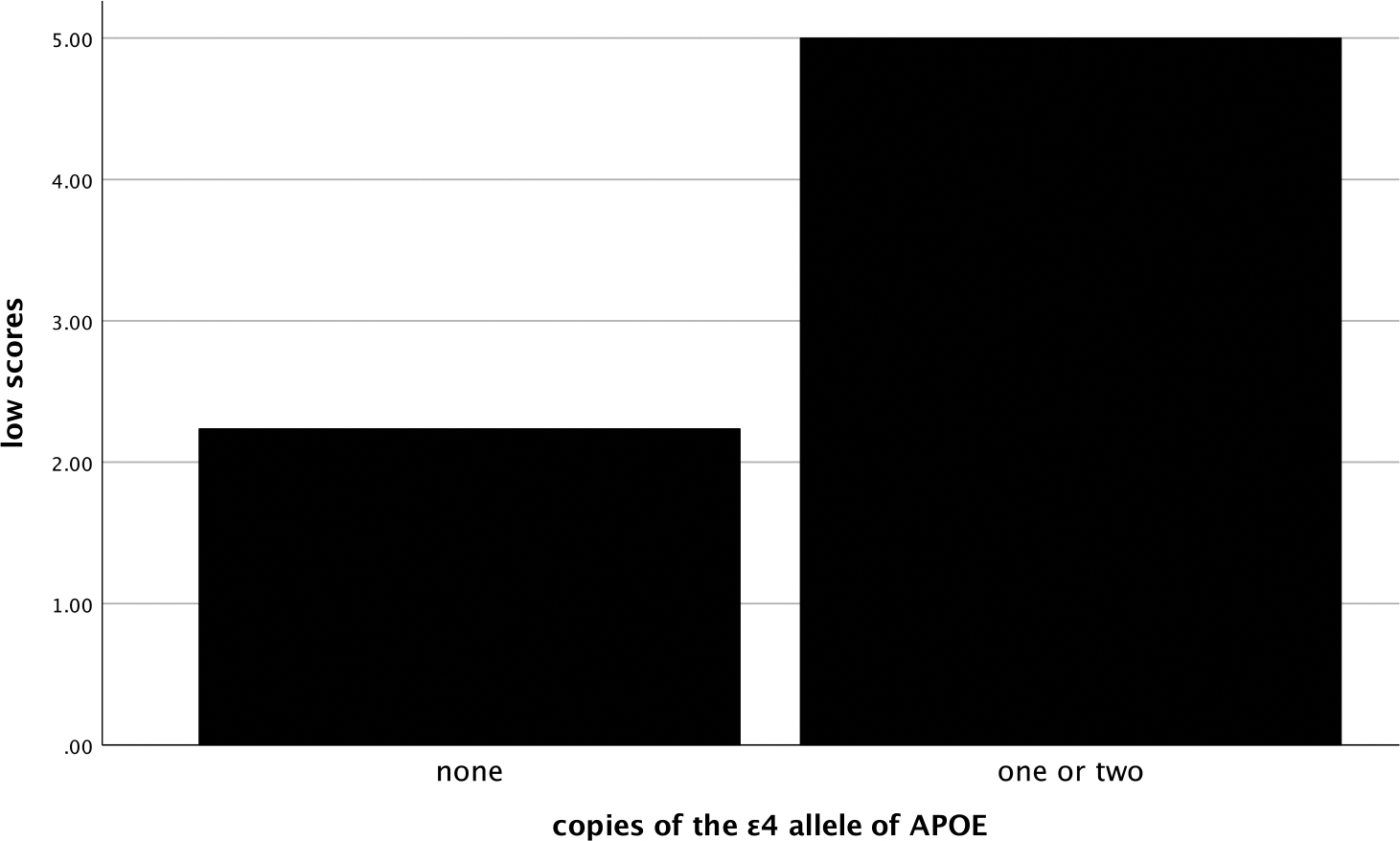
Mean number of low scores by ε4 alleles groups. *Note*: low scores = number of scores ≤ 16^th^ percentile.

In the entire sample, the number of low scores that one obtained was significantly and positively related to their SUVR (ρ=.58, *p* < .001), such that more low scores were associated with greater amyloid deposition (see Figure 1). The number of low scores was also significantly negatively correlated with hippocampal volume (ρ=−.54, *p* < .001), with more low scores being correlated with smaller hippocampi (see Figure 2). Individuals who had one or more copies of the ε4 allele of APOE had significantly more low scores on the RBANS than those with no copies of the allele (χ^2^ [2] = 34.1, *p* < .001, η^2^ = .20, see Figure 3).

**Figure 4.**
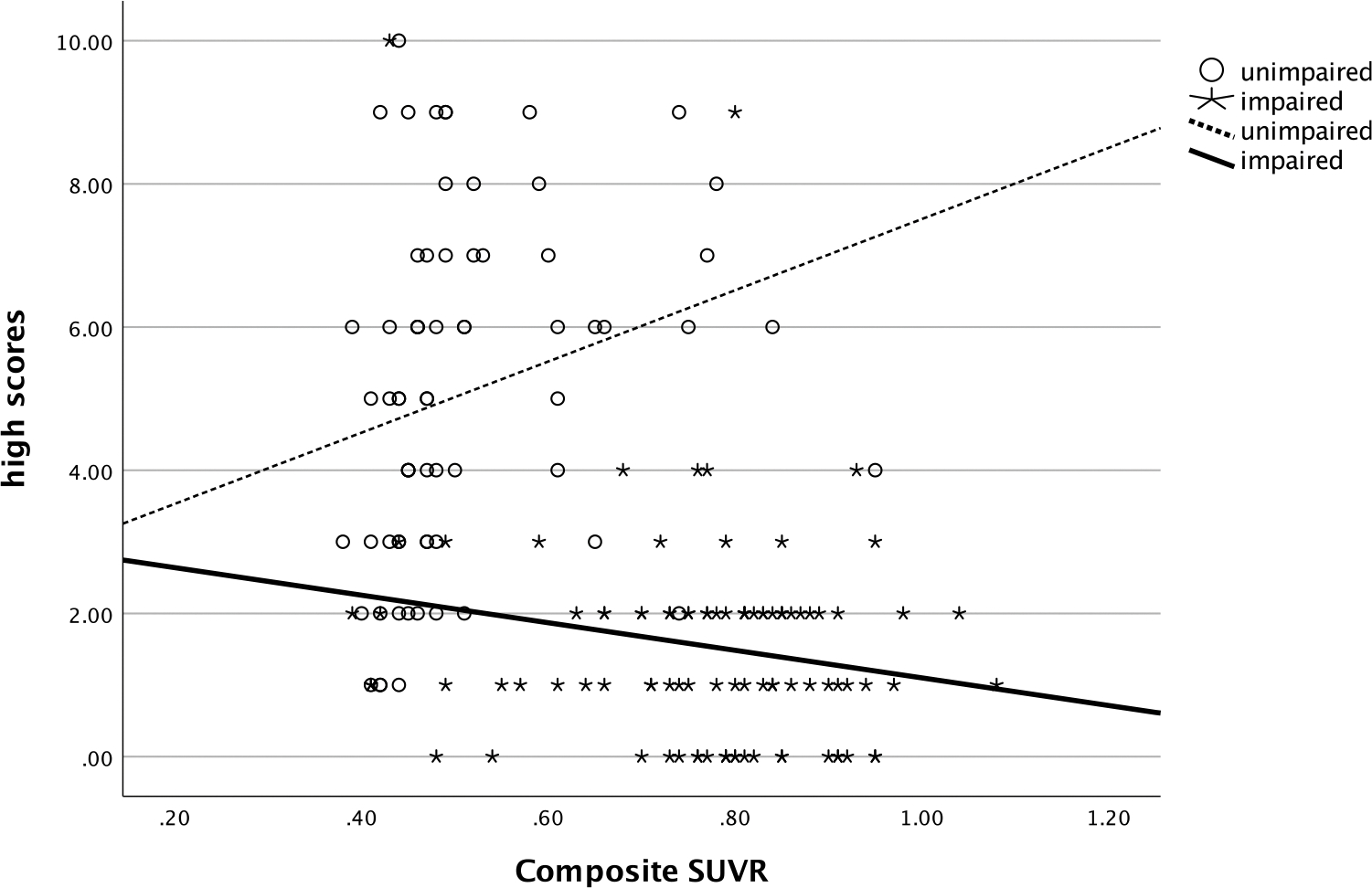
Relationship of number of high scores and amyloid deposition. *Note*: high scores = number of scores ≥ 75^th^ percentile; SUVR = standardized uptake value ratio.

**Figure 5.**
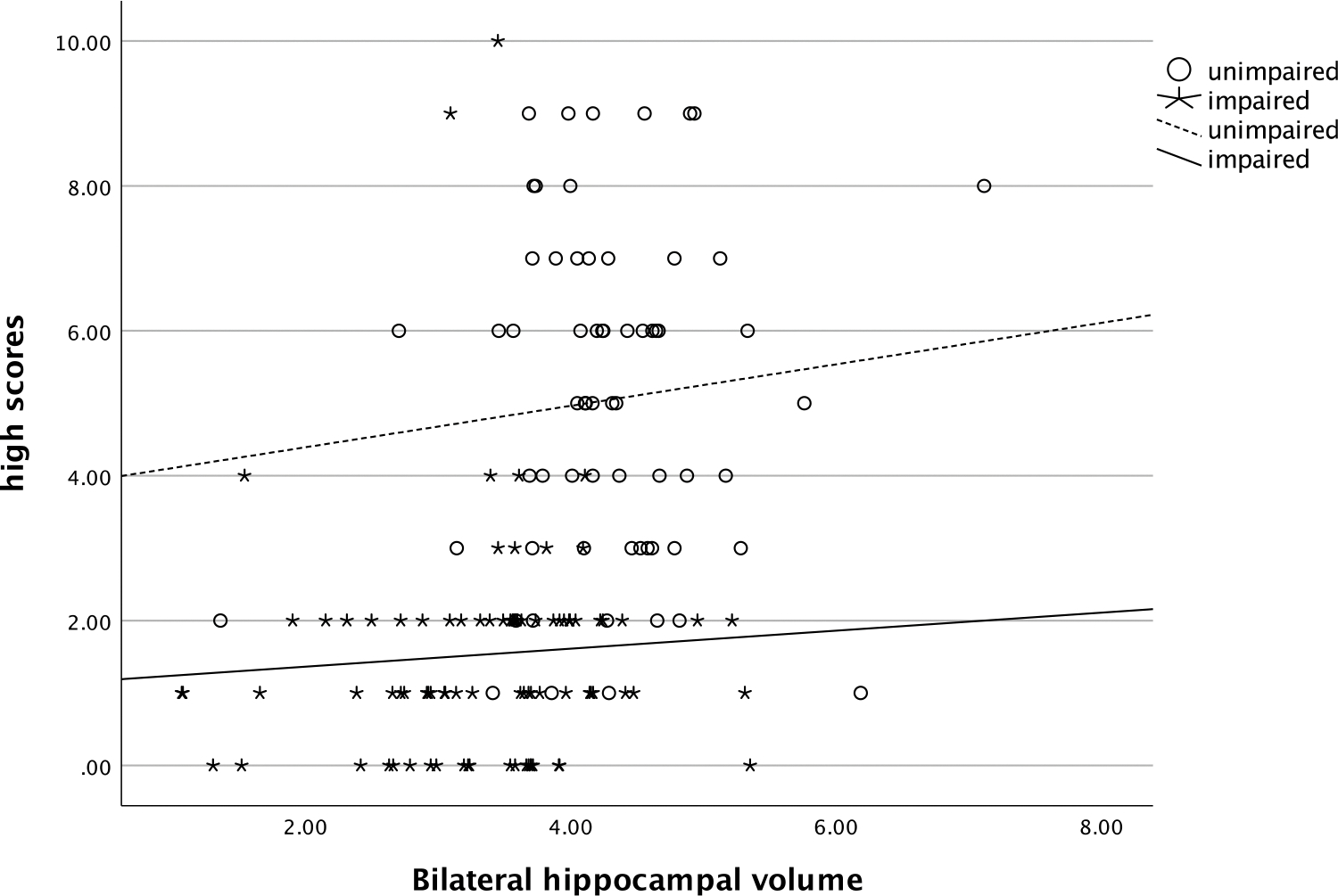
Relationship of number of high scores and bilateral hippocampal volume. *Note*: high scores = number of scores ≥ 75^th^ percentile.

**Figure 6.**
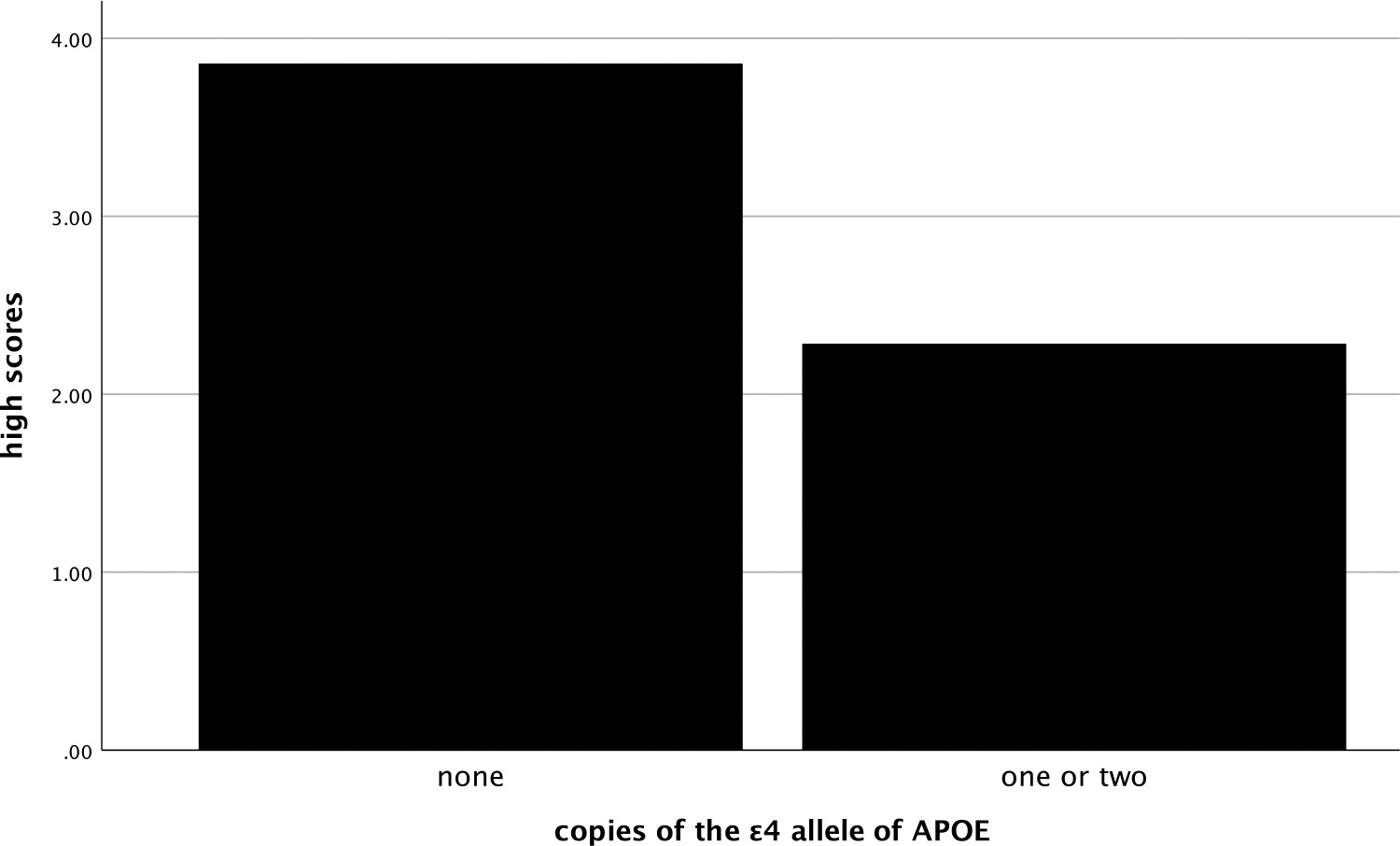
Mean number of high scores by ε4 alleles groups. *Note*: high scores = number of scores ≥ 75^th^ percentile.

In both cognitively unimpaired and impaired participants, the number of high scores was also related with these biomarkers. More high scores on the RBANS were negatively correlated with SUVR (ρ=−.44, *p*= <.001, see Figure 4) and positively correlated with hippocampal volume (ρ=.45, *p*= <.001, see Figure 5). Significantly more high scores were seen in those with no ε4 alleles compared to those with one or two copies of ε4 (χ^2^ [2] = 16.3, *p* < .001, η^2^ = .09, see Figure 6).

### Comparison with RBANS Total Scale score

**Figure 7.**
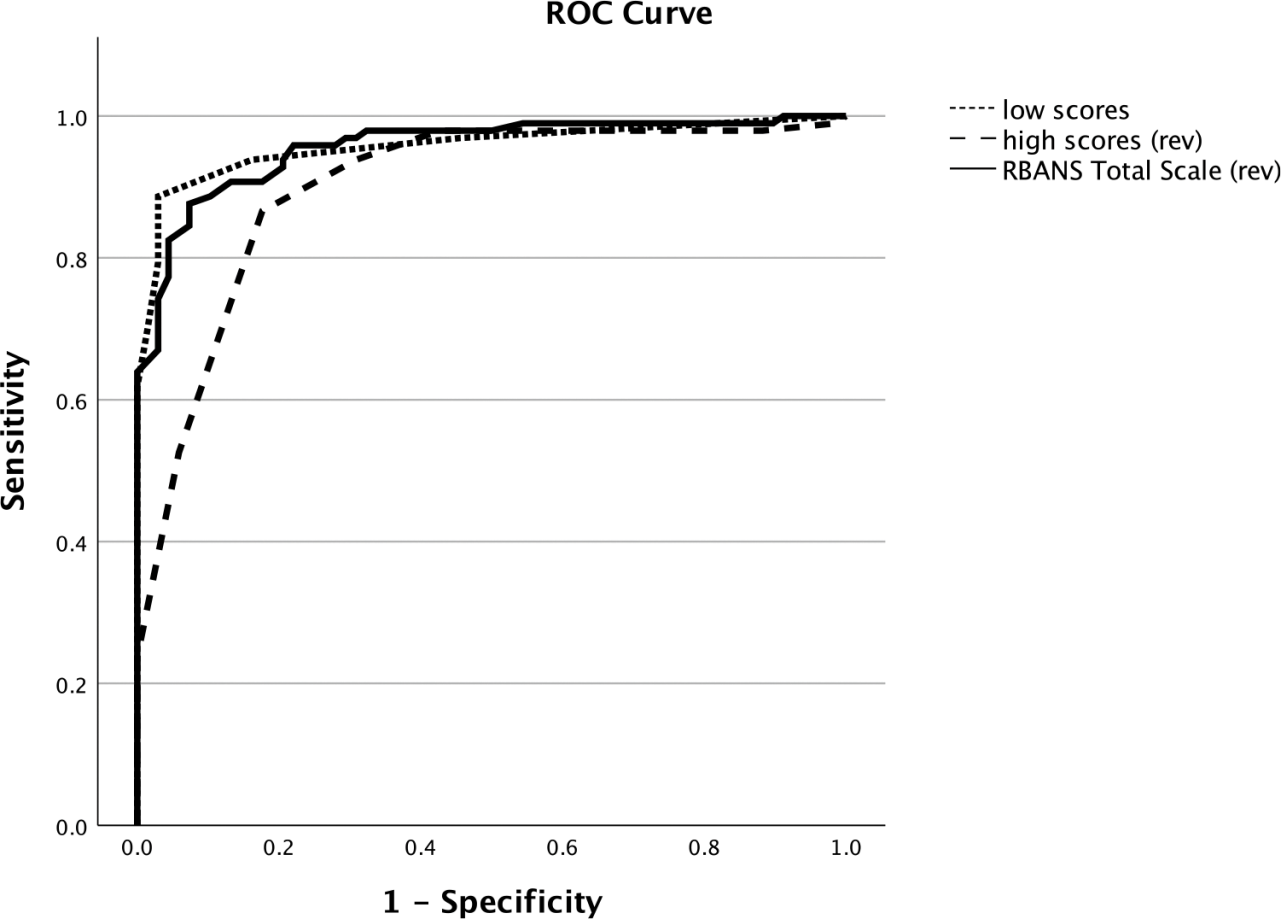
Area under the curve for identifying cognitively impaired participants with low, high, and RBANS Total Scale scores. *Note*: High and RBANS Total scores were reversed so that all three scores went in the same direction for impaired individuals.

The number of low scores identified cognitively impaired individuals with an area of the curve (AUC) in the ROC of .96. The number of high scores identified these same individuals with an AUC of .90. Finally, the RBANS Total Scale score identified the cognitively impaired individuals with an AUC of .96. These are depicted in Figure 7. AUCs for other traditional scores of the RBANS ranged from .70 (Visuospatial/Constructional Index) to .98 (Delayed Memory Index), and additional details can be obtained from the first author.

### Secondary analysis on the association with biomarkers in each cognitive group

In the cognitively unimpaired participants, there were limited associations between low/high cognitive scores and biomarkers. More low scores on the RBANS were significantly negatively related to hippocampal volumes (ρ=−.27, *p* = .028). However, more low scores were not associated with SUVR (*p* = .20) or ε4 alleles (*p* = .58). More high scores were significantly and positively correlated with SUVR (ρ=.40, *p* < .001), but this suggested that more high scores were associated with more amyloid deposition. Neither hippocampal volume (*p* = .87) nor APOE (*p* = .71) was significantly related to the number of high scores in the unimpaired participants.

In the cognitively impaired participants (i.e., MCI and AD), the greater the number of low scores was significantly and positively related to SUVR (ρ=.22, *p* = .028). The number of low scores was not related to hippocampal volume (*p* = .14) nor APOE (*p* = .11). In this impaired group, the number of high scores was not related to any of the biomarkers (SUVR *p* = .28, hippocampal volume *p* = .16, APOE *p* = .91).

## Discussion

Karr’s “other side of the bell curve” hypothesis (Karr et al., 2020) suggests that the absence of high scores on neuropsychological tests may provide valuable clinical information. Although some supportive findings related to this hypothesis have been presented (Iverson & Karr, 2021; Karr et al., 2020; Karr et al., 2024; Karr et al., 2022), a broader examination is needed. The current study attempted to expand this work in multiple ways: 1) by considering the number of low and high scores on the RBANS, 2) by considering these scores in both cognitively unimpaired or impaired participants, 3) by considering how these scores related to three common biomarkers of AD, and 4) by considering how these low and high scores compare to traditional RBANS scores. In our primary analyses in the entire sample of cognitively unimpaired and impaired individuals, high scores (i.e., ≥ 75^th^ percentile) were common in this sample on the RBANS (e.g., 86% having at least 1 of 12 high scores, which is about 7% of all possible scores [i.e., .86*.08]). Compared to the original study related to this hypothesis, Karr et al. (2020) found that 98% of a subset of the standardization sample of the Delis–Kaplan Executive Function System achieved at least one high score (or 6% of all possible scores). Using the cognitive battery of the NIH Toolbox, Iverson & Karr (2021) reported that 66% of the normative sample had at least one high score (or 9% of all possible scores). Using the Spanish-language version of the same cognitive test, Karr et al. (2022) also found that 66% got at least one high score (or 9% of all possible scores). Finally, in older adults with subjective cognitive complaints, Karr et al. (2024) observed that 94% of their sample had at least one high score (or 13% of all possible scores). As such, the percentage of high scores is similar to other studies investigating this hypothesis, even though most of the other studies used normative/standardization samples, which likely contained largely cognitive unimpaired individuals. In fact, the highest rate of high scores was in Karr et al. (2024), in which half of the sample were cognitively intact but complained of cognitive difficulties. These cognitively intact individuals with subjective complaints did have significantly fewer high test scores than their peers who did not have cognitive complaints in that study, which would be supportive of this hypothesis if the former individuals progressed to a cognitive disorder at a higher rate than the latter individuals.

Fewer studies have examined the number of low scores (i.e., ≤ 16^th^ percentile) as they relate to this hypothesis. For example, in cognitively unimpaired individuals, Iverson & Karr (2021) found that nearly 43% had at least one low score (or 6% of all possible scores). In older adults, some of whom complained of cognitive decline and others who did not, Karr et al. (2024) found that nearly 18% had at least one low score (or 3% of all possible scores). In the current cohort, low scores were more comparable to the Iverson et al., study. For example, about 75% of the entire sample had at least one low score (or 6% of all possible scores), almost half had three or more low scores, and nearly one quarter had six or more low scores. The higher frequency of low scores in the current sample seems clearly influenced by the composition of this group, which included both cognitively unimpaired and impaired older adults. And since the current study used the RBANS, which has more subtests than the other studies, there was a greater chance of finding low scores in the present study. Although the “other side of the bell curve” hypothesis originally focused on the absence of high scores, the presence of low scores is more consistent with the clinical practice of neuropsychology. In some ways, these two sets of scores (low and high) are “opposite sides of the same coin.” In the entire sample, the number of low scores was negatively related to the number of high scores. However, this was not a perfect correlation, as only about 50% of the variance of one set of scores was explained by the other set of scores. As such, both sets of scores appear to hold unique variance in explaining cognitive functioning in older adults.

One of the unique contributions of the current study was that it included both cognitively unimpaired and impaired participants. The cognitively unimpaired individuals averaged five high scores and less than one low score on the RBANS. Conversely, the cognitively impaired individuals, which contained individuals classified with amnestic MCI and mild AD, averaged 1–2 high scores and nearly six low scores on the RBANS. The number of low or high scores was significantly different between these two groups, with large to very large effect sizes. Both groups showed negative associations between their low scores and high scores, with the unimpaired individuals showing a slightly smaller relationship (ρ=−.41) than the impaired individuals (ρ=−.62, *z* = 1.79, *p* = .073). Although these findings are not surprising, such results have not been previously reported related to this hypothesis. Thus far, only Karr et al. (2024) examined this hypothesis in a more clinically relevant group than a normative standardization sample. In their study, they found that older adults with subjective cognitive complaints achieved significantly fewer high scores than their peers without cognitive complaints; however, these differences remained quite close (3.1 vs. 4.4 high scores, respectively). The larger spread of high scores between the two groups in the current study again appears expected since our impaired group had been identified (with an independent battery of tests) to show objective cognitive deficits. Nonetheless, such “process” scores (e.g., number of low and high scores) appear to provide some clinical utility in separating clinical and nonclinical cases.

The current study also sought to further examine the “other side of the bell curve” hypothesis by examining how low and high scores were associated with multiple biomarkers of AD. Since little work has been completed relating the number of low and high scores to any biomarkers, these findings were of particular interest. In the full sample, the greater number of low scores was related to greater amyloid deposition, smaller hippocampal volumes, and more copies of the ε4 allele of APOE. Conversely, in both cognitively unimpaired and impaired participants, the greater the number of high scores was associated with less amyloid deposition, larger hippocampi, and fewer ε4 alleles. As such, the number of low/high scores appear to be inversely related to AD biomarkers. In the only other study to look at this hypothesis and neuroimaging, Karr et al. (2024) failed to find an association between the number of low or high scores and any of three neuroimaging variables (i.e., regional brain volume, cortical thickness, and connectivity of the frontoparietal control network) in older adults with subjective cognitive complaints. The discrepancy between the current findings and those in the existing literature might highlight the differences between these two studies, including sample size, diagnostic groups, and biomarkers. Additional work would be needed to further understand how the number of low/high scores inform clinicians and researchers about biomarker positivity/negativity in older individuals. It is worth noting that subgroup analyses yielded fewer associations between low/high scores and biomarkers than the entire sample. Restricted range of both low/high scores and biomarker values in each group likely influenced these results.

Finally, the results of the current study did not appear to give an advantage to the use of low/high scores compared to traditional scores on the RBANS. For example, the AUC for the low scores was .96, indicating near perfect separation of the two groups based on the number of low scores. The AUC for the high scores was slightly lower at .90 but still showing good discrimination of the groups. For the RBANS Total Scale score, the AUC was .96, which was identical to that of the number of low scores. Overall, all three scores are generally comparable. Although comparable discriminations that could question if one gains much by calculating low/high scores, there may be individual patients or samples where the low/high scores and traditional measures are inconsistent, with each adding unique variance to answering the diagnostic question. In certain individual patients or samples, the low/high scores might provide complementary evidence about one’s risk for cognitive decline that is missed by traditional scores. As such, continued investigation into the clinical value of these process scores seems warranted.

The current study is not without its own limitations. First, the sample size of the current study was relatively small, especially compared to some of the other work examining this hypothesis. For example, the current study employed data from 165 older adults, whereas prior studies have utilized 250–1,050 healthy controls (Karr et al., 2020, 2021, 2022). Larger samples appear more beneficial in assessing the range of cognitive functioning, both in low and high scores. Second, the individuals in the impaired group in the current study comprised those with amnestic MCI and mild AD. As such, the current findings provide little insight into how frequently low or high scores occur in cognitive impairment due to other etiologies (e.g., frontotemporal dementia, Dementia with Lewy Bodies, vascular dementia, depression). Third, the current study used ≤ 16^th^ percentile to define low scores and ≥ 75^th^ percentile to define high scores, even though these are not opposite points along a normal distribution curve (e.g., −1.00 SD vs. +0.67 SD, respectively). However, these seem to be the ones most commonly used in the prior studies of this hypothesis. As such, these points were chosen a priori, and not to maximize the separation of the unimpaired and impaired subjects. Future studies that examine both intact and clinical groups might identify different points on the normal distribution curve to optimize that separation, which may have more clinical utility. Finally, 13 individuals did not have useable hippocampal volume data, which may have biased these relationships with low and high scores. Despite these limitations, the current work extends our understanding of the value of low and high scores in identifying cognitively unimpaired and impaired individuals. These results seem to suggest that “both sides of the bell curve” provide clinically useful information for neuropsychology.

## Supplementary Material

supplementary material

**Supplementary material.** The supplementary material for this article can be found at https://doi.org/10.1017/S1355617725101227.

## Figures and Tables

**Table 1.** Base rates of low and high RBANS index scores in the total sample and stratified by cognitive group

		Cognitive group	
	
	Total (*n* = 165)	Unimpaired (*n* = 68)	Impaired (*n* = 97)

Number of low scores			
≤ 16^th^ percentile			
12 low scores	1.2%	0.0%	2.0%
11 or more	1.8%	0.0%	3.0%
10 or more	4.8%	0.0%	8.1%
9 or more	9.6%	0.0%	16.2%
8 or more	16.7%	0.0%	28.3%
7 or more	26.2%	0.0%	44.5%
6 or more	36.9%	0.0%	62.7%
5 or more	42.3%	1.4%	70.8%
4 or more	48.3%	2.8%	79.9%
3 or more	53.7%	2.8%	89.0%
2 or more	62.0%	15.8%	94.1%
1 or more	75.7%	44.8%	97.1%
No low scores	24.4%	55.1%	3.0%
Mean (SD) of low scores	3.73 (3.30)	0.69 (0.98)	5.86 (2.62)
Number of high scores			
≥ 75^th^ percentile			
12 high scores	0.0%	0.0%	0.0%
11 or more	0.0%	0.0%	0.0%
10 or more	1.2%	1.4%	1.0%
9 or more	6.0%	11.5%	2.0%
8 or more	8.4%	17.3%	2.0%
7 or more	12.6%	27.4%	2.0%
6 or more	20.9%	47.7%	2.0%
5 or more	25.1%	57.8%	2.0%
4 or more	32.2%	69.4%	6.0%
3 or more	41.7%	82.4%	13.1%
2 or more	66.1%	94.0%	46.4%
1 or more	85.7%	100.0%	75.7%
No high scores	14.3%	0.0%	24.2%
Mean (SD) of high scores	3.01 (2.62)	5.09 (2.42)	1.54 (1.57)

## Statement of Research Significance

### Research Question(s) or Topic(s):

Recently, the “other side of the bell curve” hypothesis suggested that the absence of high scores on cognitive tests could indicate early decline in high functioning individuals. However, this hypothesis has not been examined in cognitively impaired individuals nor have the absence of high scores been compared to biomarkers of disease.

### Main Findings:

Both low and high scores were common, with unimpaired participants having more high scores and impaired individuals having more low scores. The number of low/high scores was significantly related to amyloid deposition, hippocampal volume, and copies of the ε4 allele of APOE, with each relationship going in the expected direction.
